# Measurement invariance of the GAD-5 Generalized Anxiety Disorder Scale in a Mexican general population sample

**DOI:** 10.3389/fpsyt.2022.973134

**Published:** 2022-10-10

**Authors:** Claudia I. Astudillo-García, Fernando Austria-Corrales, Leonor Rivera-Rivera, Luz Myriam Reynales-Shigematsu, José Alberto Gómez-García, Marina Séris-Martinez, Alberto Jiménez-Tapia, Rebeca Robles, Silvia Morales-Chainé, Alejandra López-Montoya, Corina Cuevas-Renaud, Filiberto Toledano-Toledano

**Affiliations:** ^1^Servicios de Atención Psiquiátrica (SAP), Secretaría de Salud, Ciudad de México, México; ^2^Comisión Nacional para la Mejora Continua de la Educación (MEJOREDU), Ciudad de México, México; ^3^Centro de Investigación en Salud Poblacional, Instituto Nacional de Salud Pública (INSP), Cuernavaca, Morelos, México; ^4^Secretariado Técnico del Consejo Nacional de Salud Mental (STCONSAME), Secretaría de Salud, Ciudad de México, México; ^5^Instituto Nacional de Psiquiatría Ramón de la Fuente Muñiz (INPRFM), Ciudad de México, México; ^6^Facultad de Psicología, Universidad Nacional Autónoma de México, Ciudad de México, México; ^7^Unidad de Investigación en Medicina Basada en Evidencias, Hospital Infantil de México Federico Gómez, Instituto Nacional de Salud, Ciudad de México, Mexico; ^8^Unidad de Investigación Sociomédica, Instituto Nacional de Rehabilitación Luis Guillermo Ibarra Ibarra, Ciudad de México, México; ^9^Dirección de Investigación y Diseminación del Conocimiento, Instituto Nacional de Ciencias e Innovación para la Formación de Comunidad Científica, INDEHUS, Ciudad de México, México

**Keywords:** anxiety, Generalized Anxiety Disorder Scale (GAD), measurement invariance, multiple-group analysis, factor analysis, statistical, mass screening

## Abstract

The primary objective of this study was to evaluate the measurement of invariance by sex, age, and educational level of an online version of the Generalized Anxiety Disorder Scale in a five-item version (GAD-5). Configural, metric, scalar, and strict invariance were evaluated using data from 79,473 respondents who answered a mental health questionnaire during the COVID-19 pandemic in Mexico. The sex variable was classified as male or female; age was categorized as minors, youth, young adults, adults, and older adults; and educational level was divided into basic, upper secondary, higher, and graduate education. To test for configural invariance, confirmatory factor models were constructed. For metric invariance, equality restrictions were established for the factor loadings between the construct and its items; for scalar invariance, equality restrictions were established between the intercepts; strict variance implied the additional restriction of the residuals. Statistical analysis was performed in R software with the lavaan package. The results show that with respect to sex, age, and educational level, configural and metric measurement invariance was confirmed (ΔCFI < 0.002; ΔRMSEA < 0.015). However, with respect to scalar and strict invariance, the results showed significant differences regarding the fit model (ΔCFI > 0.002; ΔRMSEA > 0.015). We conclude that the GAD-5 presents configural and metric invariance for sex, age, and educational level, and scalar invariance for sex and age groups. However, the scale does not demonstrate strict invariance. We discuss the implications and suggest that this result could be related to the evaluation of sociodemographic variables.

## Introduction

Anxiety disorders account for a large proportion of the global burden of disease and disability. A systematic review published in 2022 (1) reported that 301.4 million people worldwide had some type of anxiety disorder, with an age-standardized prevalence rate of 3779.5 (3181.1–4473.3) per 1,00,000 population. However, in Latin America and the Caribbean, this rate is 5502.3 (4625.9–6588.7). The global prevalence of generalized anxiety disorder (GAD) was 4.5% in 2021; although a higher prevalence has been reported in high-income countries (5.3%) than in low-income countries (2.8%), the proportion of people who have received treatment is lower in the latter (19.2 vs. 38.4%) (2). In low- and middle-income countries, most people with these disorders will never see a mental health specialist (3). It has also been reported that subthreshold anxiety disorders may have twice the frequency of the full syndrome, and are more persistent, cause greater suffering and functional impairment, and have a higher risk of onset and aggravation of other mental health conditions, such as pain and comorbid somatic disorders, increasing care costs (4).

The existing differences by sex and age must be added to this care gap. Women present greater anxiety than men. According to the 2022 GBD review, 187.5 million women suffer from anxiety disorders vs. 109.3 million men, in addition to the fact that the number of disability-adjusted life years (DALYs) increases steadily during childhood and adolescence, reaching a maximum between the ages of 25 and 34 and decreasing steadily after the age of 35 (1). In contexts such as the COVID-19 pandemic, evidence shows that there are significant differences by sex and age, with women and younger people scoring significantly higher in anxiety, and these differences are present also by educational level (5). In order to make judgments across conditions of age, sex, or educational level, scales are needed that operate equivalently for these different groups of interest (6), and that are available in non-specialized care settings.

Primary care is the ideal setting for the identification and appropriate treatment of the most common mental disorders. Screening for their early detection and treatment in primary care can improve quality of life, help contain health care costs, and limit complications from medical and mental health comorbidities (7). The application of screening scales is a useful alternative in primary care in low- and high-income countries, given existing time and resource pressures (8). These scales have the potential to improve case detection through procedures that could be incorporated into primary care practice. They direct attention to anxiety symptoms, and help to determine the current status of the individual and offer a specific diagnosis and treatment (8). Population-based screening requires that such tools have psychometric properties that allow for valid comparisons.

The factorial invariance of a scale is the statistical property that indicates whether it measures the same latent construct among the subgroups of a sample, which is a prerequisite for making valid group comparisons. The presence of non-variance could be indicative of bias due to differences in the interpretation of the items included in a scale (9). To determine whether a measure presents factorial invariance, factor loadings, intercepts, and residual variances are tested to ensure that they are equivalent in a factorial model that evaluates a latent concept. To this end, a set of increasingly restricted structural equation models are run to test whether differences between these models are significant (10). Failure to test for invariance means that different groups or subjects may respond differently to the items and that factor means cannot be reasonably compared (10).

The GAD Scale was developed as a screening tool for primary care settings (11). Its initial version consisted of nine items reflecting all of the DSM-IV diagnostic criteria for the disorder, as well as four items based on a review of existing anxiety scales (11). A seven-item version (GAD-7) has reported good to excellent sensitivity and specificity for most of the relevant DSM-5 disorders (5) in both the general population and in primary care patients (12). Measures of invariance have been reported for the GAD-7 (6, 9, 13), but not for the GAD-5, a five-item version obtained from studies of the primary care population (3, 8). The five items are directly linked to the ICD-11 diagnostic guidelines for depression and anxiety, in which a total score of 3 or more predicted 89.6% of above-threshold cases with generalized anxiety (11). This brief assessment of anxiety minimizes the time required in the patient encounter and obviates the need for paper and pencil tests and instrument scoring (3). It therefore offers a substantially more practical alternative for implementation in low-resource settings, and it may also be of considerable value in high-income countries (3).

The confirmation of parameter invariance helps to verify that the items and measures are free of biases that produce differences, which could be the result of differences in age, gender, and educational level. For example, the use of certain words may create a difference between those who fully understand an item and those who do not. In addition, gender bias in the wording of items can generate systematic error variances that may affect measurement precision. Confirming the invariance of parameters across different ages, sexes, and educational levels will help to understand whether the five attributes measured by the GAD-5 are relatively constant across groups and whether the groups analyzed share the same metric: whether the construct being measured is equivalent across groups (14). The aim of this study was thus to assess measurement invariance through the estimates of configural, metric, scalar, and strict invariance of the five-item version of the Generalized Anxiety Disorder Scale (GAD-5), across sex, age group, and educational level.

## Materials and methods

### Participants and procedure

We used a convenience sampling strategy to recruit 79,473 people who were analyzed for this study. Participants answered the GAD-5 questionnaire from April 1 to December 31, 2020, as part of the survey Atención Psicológica a Distancia para la Salud Mental por la contingencia por COVID-19 (Remote Mental Health Care during the COVID-19 Pandemic). This survey was part of the Mexican effort, led by the Secretary of Health, the Universidad Nacional Autónoma de México (UNAM), the Instituto Nacional de Psiquiatría, and civil society organizations to meet the mental health needs of the population and reduce the stress caused by the pandemic. The survey was administered by a team from the UNAM Faculty of Psychology through the federal government's coronavirus.gob.mx website. On this website, people were invited to participate voluntarily and confidentially and offered care resources according to the risk levels detected for different mental health problems. The questionnaire was self-administered online. A description of the survey and the variables assessed is available in a previous publication (15).

### Study variables

The sociodemographic variables considered were sex, age group, and educational level. Sex was classified as male or female. Age was categorized as minors (13–17 years), youth (18–25 years), young adults (26–35 years), adults (36–59 years), and older adults (60 years and older). Educational level was divided into basic (elementary and junior high school), upper secondary (high school or equivalent), higher education (undergraduate degree) and graduate (specialty, master's, and doctoral degrees). The age categories are consistent with Mexican law that considers adulthood to begin at age 18 and senior citizens to be those over 60. The intermediate ages were divided into three groups that represent the life trajectories of adults in Mexico. However, it should be noted that the complexity of life trajectories makes it difficult to construct a universal division of different life stages (16). The categories of educational level were based on the organization of the educational system in Mexico, which includes basic (elementary and junior high school), middle (high school), and higher education (university); the latter was divided into separate categories for undergraduate and graduate education.

The GAD-5 consists of five items: “I feel nervous, anxious, or about to burst,” “I have felt unable to control my worries,” “I have felt so worried, I have been unable to keep still,” “I have found it hard to relax,” and “I have felt afraid that something terrible was going to happen.” Participants were asked to what extent each of these items described them in the past 2 weeks. The standard response form was modified to match the rest of the instruments used in order to avoid having to provide different instructions and response options for each part of the questionnaire. The response options for the entire survey were a 10-point Likert scale, where 0 indicated “does not describe me” and 10 “describes me exactly.” With five items, the range of possible scores was thus 0–50 points. There is evidence suggesting that increasing the number of response options increases validity coefficients by 0.04 (17). This evidence also suggests that the coefficients do not rise artificially as the number of response options increases; however, the validity does consistently improve. In another study, Alwin (18) conducted a confirmatory factor analysis to compare the performance of the versions with seven and eleven response options and found that the latter had better validity and reliability and lower invalidity indices.

### Data analysis

We first performed a confirmatory factor analysis (CFA), using R software and the lavaan package (19), to test the theoretical structure of the scale as well as its unidimensionality. The covariance matrix was analyzed using the maximum likelihood method, applying the Satorra–Bentler correction (20), since the data do not assume multivariate normality. The fit of the model was assessed with four fit indices. The comparative fit index (CFI) takes possible values between 0 and 1, with a value of at least 0.90 denoting adequate fit and a value greater than or equal to 0.95 a very good fit. The Tucker-Lewis index (TLI) also has a range from 0 to 1 with the same interpretation criteria. The Root Mean Square Error Approximation (RMSEA) should ideally have values of <0.06, although values of 0.08 are considered acceptable. Finally, the Standardized Root Mean Square Residual (SRMR) is considered acceptable with a value <0.10 and a good fit with a value <0.05 (21).

We next assessed measurement invariance using multi-group confirmatory factor analysis; this technique makes it possible to gradually impose restrictions in order to test different levels of parameter invariance: configural, metric, scalar, and strict. The first step was to test configural invariance; this model was used as a baseline for comparison with models that gradually incorporated more equality constraints. To assess configural invariance, it was necessary to keep the factor loading structure constant between the different comparison groups, although the values of the loadings, factor variances, and covariances could vary because they were not restricted to being equal. Metric invariance was subsequently determined by establishing equality restrictions on the values of the factor loadings. We then proceeded to test scalar invariance through the establishment of equality restrictions between the intercepts, and finally strict invariance, where equality was also restricted among residuals. We evaluated changes in the comparative fit index (CFI) to assess the measurement invariance between the different groups: a change in CFI of −0.01 or more from the baseline was used to reject the between-group invariance hypothesis (22). We also evaluated ΔSRMR and ΔRMSEA as alternative fit indices, as suggested by Chen (23).

## Results

Data were analyzed from 79,473 people who participated voluntarily and answered the questionnaire. The sample included 60.79% women and 39.21% men, with an average age of 35.11 years (*SD* = 12.74). The distribution by age group and educational level is shown in Table 1.

**Table 1:** Sociodemographic characteristics of the sample.

**Variable**	***n* (%)**
**Sex**
Women	48,308 (60.79)
Men	31,165 (39.21)
**Age group**
Minors	6,392 (8.04)
Youth	14,967 (18.83)
Young adults	22,267 (28.02)
Adults	32,760 (41.22)
Older adults	3,087 (3.89)
**Educational level**
Basic education	11,703 (14.73)
Upper secondary education	23,444 (29.50)
Higher education	35,318 (44.44)
Graduate	9,008 (11.33)

### GAD-5 factor analysis

**Figure 1:**
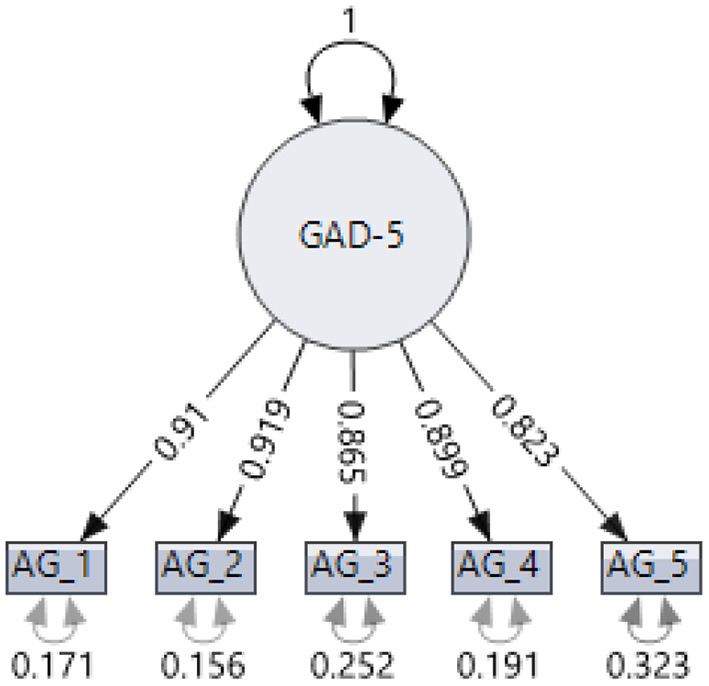
Results of the confirmatory factor analysis of the GAD-5.

The resulting model showed an adequate fit between the theoretical model and the empirical data, as shown by the following fit indices: CFI = 0.993; TLI = 0.987; RMSEA = 0.07, CI [0.075, 0.081]; SMRM = 0.009. Table 2 shows the factor loads of items in the GAD-5. The resulting model, as well as the standardized parameters, can be seen in Figure 1.

**Table 2:** Factor loads of GAD-5 items.

**Item**	Standardized coefficient (β)^*^
I feel nervous, anxious, or about to burst	0.910
I have felt unable to control my worries	0.919
I have felt so worried I have been unable to keep still	0.865
I have found it hard to relax	0.899
I have felt afraid that something terrible was going to happen	0.823

* All values are significant, p < 0.001.

### Multi-group CFA and measurement invariance

Once the unidimensionality of the GAD-5 and its parametric stability were demonstrated, variances were divided by sex, age group, and educational level, according to the categorizations described above. Equality restrictions were then gradually imposed, using the configural model as the baseline.

As regards invariance by sex, the configural invariance showed a good fit with respect to the general model, indicating a lack of significant differences in the factorial structure between women and men. When equality restrictions were placed on the factor loadings (metric invariance), no differences were observed in the comparative fit index (ΔCFI = 0.000). This evidence suggests that the GAD-5 is metrically invariant by sex. Equality restrictions were then imposed on the intercepts (scalar invariance), reducing the ΔCFI by −0.001, suggesting a lack of significant differences. Finally, after imposing equality restrictions on residuals (strict invariance), a change of −0.008 was observed in the ΔCFI, a value of <0.01, the traditional criterion for assessing the invariance of parameters. As regards age, five groups were compared: minors, youth, young adults, adults, and older adults. Table 3 shows that differences in the ΔCFI in the metric, scalar, and strict invariance are in all cases less than the criteria established by Cheung and Rensvold (22), suggesting that the GAD-5 is invariant at the configural, metric, scalar, and strict levels. In relation to educational level, we observed that changes in the ΔCFI in metric, scalar, and strict invariance do not exceed the −0.01 criterion, suggesting that the GAD-5 is invariant across educational levels. The results are shown in Table 3.

**Table 3:** Results of tests of measurement invariance.

**Model**	***X* ^2^**	***df***	**CFI**	**TLI**	**SRMR**	**RMSEA**	**Model comparison**	**ΔCFI**
**By sex**
Configural	997.856	10	0.993	0.987	0.010	0.079 (0.074-0.083)	-	-
Metric	1,223.088	14	0.993	0.990	0.013	0.068 (0.065-0.071)	Configural – Metric	0.000
Scalar	1,659.291	18	0.992	0.991	0.015	0.065 (0.063-0.068)	Metric – Scalar	−0.001
Strict	3,180.108	23	0.984	0.986	0.020	0.082 (0.080-0.085)	Scalar – Strict	−0.008
**By age**
Configural	1,126.013	25	0.993	0.986	0.010	0.082 (0.078-0.086)	-	-
Metric	1,646.677	41	0.992	0.990	0.020	0.069 (0.066-0.072)	Configural – Metric	−0.001
Scalar	2,190.414	57	0.991	0.992	0.022	0.063 (0.063-0.065)	Metric – Scalar	−0.001
Strict	3,165.974	77	0.983	0.989	0.023	0.073 (0.071-0.075)	Scalar – Strict	−0.008
**By educational level**
Configural	959.343	20	0.994	0.987	0.009	0.077 (0.073-0.081)	-	-
Metric	1,194.531	32	0.994	0.992	0.011	0.061 (0.058-0.064)	Configural – Metric	0.000
Scalar	2,054.791	44	0.991	0.991	0.017	0.063 (0.061-0.066)	Metric – Scalar	−0.003
Strict	2,713.069	59	0.986	0.990	0.019	0.067 (0.065-0.069)	Scalar – Strict	−0.005

To confirm these results based on the traditional criteria for assessing the invariance of parameters, the change in CFI (ΔCFI), additional assessments were made using two alternative indices suggested by Chen (23): changes in the RMSEA of 0.015 and the SMRM of 0.030 for metric invariance, and changes in the scalar and strict invariance of 0.015. The results are summarized in Table 4 for each of the comparison variables: sex, age group, and educational level.

**Table 4:** Alternative fit indices to evaluate measurement invariance by sex, age, and education.

**Model**	**SRMR**	**RMSEA**	**Model comparison**	**ΔCFI**	**ΔSRMR**	**Δ RMSEA**
**By sex**
Configural	0.010	0.079 (0.074–0.083)	-	-	-	-
Metric	0.013	0.068 (0.065–0.071)	Configural – Metric	−0.001	0.004	−0.011
Scalar	0.015	0.065 (0.063–0.068)	Metric – Scalar	−0.001	0.002	−0.002
Strict	0.020	0.082 (0.080–0.085)	Scalar – Strict	−0.008	0.004	−0.017
**By age**
Configural	0.010	0.082 (0.078–0.086)	-	-	-	-
Metric	0.020	0.069 (0.066–0.072)	Configural – Metric	0.000	0.011	−0.013
Scalar	0.022	0.063 (0.063–0.065)	Metric – Scalar	−0.001	0.001	−0.006
Strict	0.020	0.082 (0.080–0.085)	Scalar – Strict	−0.008	0.002	0.010
**By educational level**
Configural	0.009	0.077 (0.073–0.081)	-	-	-	-
Metric	0.011	0.061 (0.058–0.064)	Configural – Metric	0.000	0.002	−0.015
Scalar	0.017	0.063 (0.061–0.066)	Metric – Scalar	−0.003	0.006	0.002
Strict	0.019	0.067 (0.065–0.069)	Scalar – Strict	−0.005	0.003	0.004

The results by sex and age showed that ΔSRMR and ΔRMSEA have values of <0.030 and 0.015 respectively in assuming metric and scalar invariance, suggesting that these invariances might be present, but not strict invariance. However, the values observed for ΔRMSEA indicate significant differences in the model, so this possibility is not empirically supported. As for educational level, there is only metric, not scalar or strict invariance, since the ΔRMSEA value is −0.015.

Taken together, these findings suggest that the GAD-5 has psychometric properties that provide invariant measurements for the sociodemographic characteristics of sex, age, and educational level. However, the invariance is not complete in all cases. The traditional ΔCFI and alternative indexes of ΔSRMR and ΔRMSEA coincide to show the following: (a) by sex, GAD-5 has configural, metric, and scalar invariance; (b) by age group, it has configural, metric, and scalar invariance; and (c) by educational level, it has configural and metric invariance.

## Discussion

Using data drawn from a large Mexican general population sample, we assessed measurement invariance of the GAD-5 by sex, age, and educational level. Our findings indicate that the GAD-5 conforms to the proposed theoretical structure, since a unidimensional construct of generalized anxiety symptomatology was obtained, which presented configural and metric invariance in the comparison by sex, age, and educational level, and scalar invariance in the comparison by sex and age. This provides evidence that the use of the GAD-5 as a screening instrument in the general population allows for adequate comparisons between men and women and between age groups.

The results of the measures of configural, metric, and scalar invariance, both by sex and by age group, show that the construct (factor loadings) and the levels of the underlying items (intercepts) are equal in all the groups tested. Accordingly, these groups attribute the same meaning to the latent construct studied, and their scores on the latent variable can be compared. Although strict variance was not achieved, indicating that the explained error variances are not equal in all groups, they can still be compared with respect to the latent variable. It should be noted that the latent variable is measured with different degrees of error between groups (10). However, provided that at least two loadings and intercepts are the same across groups, valid inferences can be made about the differences between the means of the latent factors in the model (10).

Since there is still a significant debate concerning the fit indices to be used to assess parameter invariance, this study used traditional indices (ΔCFI) and alternative indices that have been proposed in recent years (ΔSRMR and Δ RMSEA) to obtain additional evidence. It was therefore possible to observe that some scalar invariance hypotheses were rejected when more than one fit index was compared. Likewise, we should note that the confirmation of certain measurement invariance hypotheses does not mean there are no variations between the attributes of the different groups under comparison. What it means is that the instrument is able to efficiently measure, and with less error, between the different groups, without affecting the measurements, which increases the internal validity of the inferences that can be drawn. The results showed, for example, that the hypotheses of configural invariance and metric invariance are sustained across educational levels, whereas the scalar and strict hypotheses are rejected. This evidence suggests that the anxiety characteristics measured by the GAD-5 are present at all four educational levels (configural invariance) and that the metric for measuring anxiety in each level is identical (metric invariance). However, the latent averages (intercepts) obtained from the measurements between the different levels vary significantly, as does the degree of error in the estimation process (residuals).

At the same time, it is important to recognize that although measures of configural, metric, scalar, and strict invariance are enormously useful in the construction and evaluation of psychological theories, their validity and existence in the real world of psychological measurement and research can never be definitively established in practice: they remain more of an ideal (24). The challenge for researchers who allow for partial invariance (in other words, that evidence is not obtained for all types of invariance) is to determine how much non-invariance can be tolerated while still claiming to measure the same construct across groups: they must make a decision based on the anticipated threat to the validity of their findings in each course of action (25). Novel approaches have been proposed for the use of partial invariance analysis through simulations, and it has been suggested that these can outperform total and partial invariance approaches when there are many small differences in item parameters (26).

Despite these considerations, the GAD-5 is a useful alternative in the general population that can be used in primary care settings, like the GAD-7 (11, 12, 27–31), and during health emergencies such as the COVID-19 pandemic. In this respect, the GAD-5 offers the practicality of web-based application in addition to the novelty of the response format used. These features contribute to the current debate on how the number of response options affects the psychometric properties of Likert-type scales (32, 33): it has been reported that reliability increases and excessive interpolation is avoided when response options increase from five to seven (34, 35), a result that could be more evident in online surveys.

Finally, it is important to consider the need to identify anxiety-like symptomatology even if it has only been present for a short time, and the GAD-5 refers to the previous 2 weeks. Short periods of anxiety have been reported to be predictive of subsequent psychopathology and may present as much associated disability at 6-month follow-up as longer periods (3). Including these screening options in routine care settings could therefore be a highly effective preventive action for the detection of common mental disorders in primary care, and improve the level of detection and diagnosis of these disorders in public health systems (3, 36).

### Limitations

Although our data represent a robust sample of the Mexican population, it should be noted that data collection was conducted entirely online, which may lead to participation as well as information bias. At the same time, by considering only the categories of male and female, we omitted transgender, nonbinary, and gender-diverse individuals, who experience more mental health issues than their cisgender peers, including higher rates of depression, suicide, violence, and drug use (37). By achieving parameter invariance in these groups, we could confirm whether variations are due to the level of anxiety presented by the person, irrespective of group membership. There is thus a need to obtain scientific evidence regarding this sexually diverse population to support its mental health by strengthening the competencies of health system professionals, and also for the formulation of public policy (38).

We must also recognize that cross-sectional measurement does not allow for the exploration of invariance over time, which is also important (39). To do so, it would be necessary to conduct follow-up measurements to assess long-term effects in the population, which was beyond the initial scope of the mental health strategy during the COVID-19 pandemic. Future studies could evaluate the partial invariance of the GAD-5 parameters at levels that could not be confirmed in this study, for example at the educational level, and for scalar and strict invariance in all cases. We also think it is important to evaluate other variables of interest, but given that our study was a secondary analysis, this was not possible. Finally, the absence of additional validation criteria and comparative studies of the validity and usefulness of the GAD-5 could also be considered a limitation of the study requiring future research.

Despite these limitations, our results show that the scale performs quite satisfactorily, and this allows us to make several observations. First, it is possible to use the scale without the need for any special adjustment or scoring to detect anxiety levels in the population, in contrast to other measures that are used indiscriminately without knowing their psychometric properties or whether they require specific scoring to accurately place examinees on a continuum. Second, the scale allows for comparisons between examinees, regardless of their age, educational level, or sex, since the data show invariance across these variables, facilitating direct comparisons without the need for linear transformations to compare populations. Third, the five attributes measured by the GAD-5 are sufficiently general as to be present in all of the groups compared, which in itself constitutes evidence of external validity.

## Conclusion

The GAD-5 shows a unidimensional theoretical structure and configural, metric, and scalar invariance in its comparisons by sex and by age group, which supports its use as a screening instrument in the general population. Since it is a short, easily administered instrument, its use could make a crucial contribution to the identification and treatment of mental health problems in both the general population and the primary care setting. This study adds to the growing evidence about the concise and simple GAD-7 questionnaire, demonstrating that its five-item version, the GAD-5, could facilitate its application in primary care settings. The brevity and predictive value of this scale suggest its potential value as an initial assessment tool for clinicians that facilitates timely intervention to treat these disorders.

## Data availability statement

The data presented in this study are available on request from the corresponding author.

## Ethics statement

The study was reviewed and approved by the Research Ethics Committee of the UNAM Psychology Faculty, registration number FPSI/422/CEIP/157/2020. All data are protected under the computer security standards of Mexican personal data protection laws. The participants provided their written informed consent to participate in this study.

## Author contributions

CA-G and FA-C: conceptualization, methodology, and formal analysis. CA-G, FA-C, LR-R, LR-S, JG-G, MS-M, AJ-T, and FT-T: research. RR, SM-C, AL-M, and CC-R: data curation. CA-G, FA-C, and AJ-T : writing of original draft. All authors participated in the writing, review, editing, read and approved the final manuscript.

## Funding

This work presents some results of the research project HIM/2015/017/SSA.1207 Effects of mindfulness training on psychological distress and quality of life of the family caregiver, Main researcher: Filiberto Toledano-Toledano, Ph.D. The present research was funded with federal funds for health research and was approved by the Commissions of Research, Ethics and Biosafety [Comisiones de Investigación, Ética y Bioseguridad], Hospital Infantil de México Federico Gómez, Instituto Nacional de Salud. The funding agency had no control over the design of the study; the collection, analysis and interpretation of the data; or the writing of the manuscript.

## Conflict of interest

The authors declare that the research was conducted in the absence of any commercial or financial relationships that could be construed as a potential conflict of interest.

## Publisher's note

All claims expressed in this article are solely those of the authors and do not necessarily represent those of their affiliated organizations, or those of the publisher, the editors and the reviewers. Any product that may be evaluated in this article, or claim that may be made by its manufacturer, is not guaranteed or endorsed by the publisher.
